# Synthesis, Photochemical and Photoinduced Antibacterial Activity Studies of *meso*-Tetra(pyren-1-yl)porphyrin and its Ni, Cu and Zn Complexes

**DOI:** 10.3797/scipharm.1003-13

**Published:** 2010-08-07

**Authors:** Tamara Zoltan, Franklin Vargas, Carlos Rivas, Verónica López, Jhackelym Perez, Antonio Biasutto

**Affiliations:** 1 Laboratorio de Fotoquímica, Centro de Química, Instituto Venezolano de Insvestigaciones Científicas, I.V.I.C., Apartado 21827, Caracas 1020-A, Venezuela; 2 Universidad de Carabobo, Facultad Experimental de Ciencia y Tecnología, Escuela de Química, Valencia, Edo. Carabobo, Venezuela

**Keywords:** Pyrenylporphyrin, Singlet oxygen, Fluorescence, Photo-oxidation

## Abstract

The synthesis of the *meso*-tetra(pyren-1-yl)porphyrin (**1**) was successfully accomplished by means of the pyrrole condensation with pyrene-1-carb-aldehyde in acidic media. Its metallization was carried out in an almost quantitative yield to obtain the corresponding complexes of Ni(II) (**2**), Cu(II) (**3**) and Zn (**4**). Their photophysical properties such as fluorescence quantum yield and energy transfer to oxygen for an efficient generation of singlet oxygen were determined. Their photophysical and photochemical properties were compared with those of other similar porphyrin derivatives such as tetraphenylporphyrin and tetranaphthylporphyrin. Photochemical studies on their effectiveness as photosensitizer were carried out by means of the photoinduced oxidation of aromatic alcohols like α-naphthol to naphthoquinone. The antibacterial photoactivity assay for compounds **1**–**4** was testeted against *Escherichia coli* (ATCC 8739) and its proliferation and viability were measured by chemiluminescence. An efficient inactivation of E. *coli* was observed. This was more efficient for compounds **2** and **3**, following the direct relationship to high generation of singlet oxygen by these compounds.

## Introduction

The most important family of photosensitizers for the production of singlet oxygenderives from porphyrins and chlorophylls, originating high singlet oxygen yields (^1^O_2_). Singlet molecular oxygen plays an important role in both natural and artificial photochemical processes. It is generally assumed that ^1^O_2_ can be generated *via* photosensitized reactions. The mechanism of the photosensitized reactions begins with the formation of the triplet state photosensitizer and energy transfer to triplet oxygen leading to ^1^O_2_ formation [1–6].

The interaction of porphyrin derivatives with oxygen under the influence of light, as well their photostability, has been a matter of great interest. In fact, the use of these compounds in the field of photodynamic therapy [6, 7] as well as photo-oxidation sensitizers stimulating their use the highly efficient intersystem crossing to its triplet state and its long life time which in turn is responsible for the high quantum yield of singlet molecular oxygen. However, various other applications for such compounds, in the presence of light, outside the medical domain demand that the persistence of the porphyrin be critically tied to a minimal challenge by singlet oxygen, an especial stability toward singlet oxygen. This prompted us to carry out a study on the photochemically excited estates of synthetic tetra-arylporphyrins and their metallic complexes, for the optimization of photoinduced oxidation of organic compounds.

On the other hand, with porphyrins, an oxidative attack involving the *meso*-positions [10–11] relies on steric accessibility [12], and one can as the question whether this vulnerability is further decreased by the introduction of appropriate substituents (vulnerability in this sense would be a composite parameter, increasing with both Φ^1^O_2_ and the ease of attack on macrocycle by ^1^O_2_.

The present work aims at the study of the photoinduced generation of reactive species of oxygen (ROS, singlet oxygen and superoxide ion) by means of *meso*-tetra(pyren-1-yl)porphyrin (TPyP, **1**) and metallo-tetrapyrenylporphyrins NiTPyP (**2**), CuTPyP (**3**) and ZnTPyP (**4**) compounds to be used as sensitizers. This work has as it bases, besides its synthesis, the effects on the absorption and emission bands on the photosensitized generation of the ROS, through the substitution of the phenyl rings in tetraphenylporphyrin (TPP) by pyrene groups. On the other hand, singlet oxygen (O_2_(^1^Δ_g_)), a short-lived, highly oxidative cytotoxic species has received remarkable attention from chemists and biochemists for its interesting mechanistic and synthetic aspects as well as for important environmental, chemical, biological and medical significance.

It is important to stand out that until now studies of this type of porphyrins in their free basic form and complex of gold have been reported only by Knör, as an example in the studies of photosensitized oxidation of guanine and photocleavage of DNA [13, 14].

One of the main global tendencies at the present time is that of developing friendly processes to the atmosphere to substitute technical and previous industrial processes, which don’t take into consideration the impact that reagents, products and/or reaction conditions exert indiscriminately on the environment. The use of photochemical resources, such as the oxidizing agent ^1^O_2_, as one of the alternatives to control the proliferation of chemical contaminants, particularly from the pharmaceutical and industrial areas and from other kinds of waste, which derive from chemical agents, is becoming today an important area of research to solve this accruing problem.

The photosensitized generation of singlet oxygen can be used for oxygenation of variable intensity as a function of Φ^1^O_2_ in the process of degradation of organic or biological compounds in residual water and also as new alternatives for photo-inactivation of micro-organisms [15–19]. In this way we propose and used a model system for the sensitive photo-oxidation of an aromatic alcohol, α-naphthol to naphthoquinone by compounds **1** to **4**, verifying in the course of this work the efficiency of this conversion.

## Experimental

### Chemicals and Materials

All solvents either analytical or HPLC grade were obtained from Merck (Darmstadt, Germany). Their purity was 99.2% as determined by ^1^H NMR-spectroscopy (Brucker Aspect 3000, 300 and 500 MHz) and UV-Vis spectrometry (Lambda 650 spectrophotometer, Perkin Elmer). The fluorescence spectra and the quantum yields were registered with a Shimadzu RF 1501 spectrofluorophotometer and also with a PerkinElmer LS Luminescence spectrometer.

The structures of the isolated products were elucidated by ^1^H NMR and ^13^C NMR (Brucker Aspect 3000, 300 and 75 MHz respectively), I.R. (Nicolet DX V 5.07) and mass spectra Finnigan TSQ Quantum Ultra (Thermo Electron Corporation, Massachusetts, USA). Elemental analyses were performed by “Laboratorio Nacional de Análisis Químico” – IVIC – Centro de Química, Caracas, Venezuela in a Fisions Instrument EA-1108. pyrene-1-carbaldehyde, pyrrole, propionic acid, Zn-, Cu- and Ni-acetate, rhodamine B, 3-aminophthalhydrazide (Luminol), 1-(4,5-dimethylthiazol-2-yl)-3,5-diphenylformazan, tetraphenylporphyrin (TPP), 1-naphthol, naphthoquinone and 9,10-diphenylanthracene were purchased from Sigma-Aldrich (St. Louis, MO, USA). UV-Vis spectrophotometry of the tetrapyrenylporphyrin (TPyP) and metallotetrapyrenyl porphyrin (MTPyP) solutions was followed using a Perkin Elmer Lambda-35 UV-Vis spectrophotometer (USA).

### Synthesis and characterization of the TPyP and metalized Porphyrins

*Meso*-tetra(pyren-1-yl)porphyrin (TPyP, **1**) was synthesized according to the following method: pyrene-1-carbaldehyde (0.5 × 10^−3^ mol) and an equimolar quantity of pyrrole were added simultaneously to refluxing propionic acid (0.5 l) and the mixture was refluxed for 1 hour before being allowed to cool and stand at room temperature overnight. The product was filtered and washed with water and methanol to give purple crystals (yield 45%). The purification of each one of the compounds required more than one separation in preparative and column chromatography (CH_2_Cl_2_/MeOH, 3:1). The absorption, emission, ^1^H-, ^13^C-NMR and EPR spectra were compared with those reported in the literature [20–23]. The metalloporphyrins **2** (TPyPNi), **3** (TPyPCu) and **4** (TPyPZn) were synthesized by means of the following method: to the TPyP (300 mg) in boiling CHCl_3_ (100 ml) was added a saturated solution of the metal-acetate in methanol (0.02 M, 1 ml). After a 12 hours refluxing and following the reaction by UV-Vis-spectrophotometry, the mixture was concentrated, diluted with a little methanol, and after cooling the metal-complex was filtered off and isolated in virtually quantitative yield.

### Quantum yields

Quantum yields of fluorescence were determined for compounds **1**–**4**. The relative quantum yields of fluorescence at room temperature were determined by comparing the corrected fluorescence intensity of compounds **1**–**4** with that of the following compounds: *meso*-tetra(pyren-1-yl)porphyrin in chloroform Φ_F_ = 0.110 and rhodamine B (at a concentration of 1.0 × 10^−6^ M, Φ_F_=0.69/ethanol), and with that of quinine bisulphate in 0.05 M H_2_SO_4_ (fluorescence quantum yield, 0.55) or pyrene (Φ_F_ = 0.38 / CH_2_Cl_2_) [24–27].

For the quantum yield determinations the photolysis was allowed to proceed to less than 10% product formation to minimize light absorption by the photoproducts and additional products from side reactions. The photon flux incident on 3 ml. of solution in quartz cuvettes of 1 cm optical path was measured by means of a ferric oxalate actinometer and it was of the order of 10^15^–10^16^ quanta s^−1^.

### Singlet oxygen (^1^O_2_) generated in cell-free systems; diphenylanthracene test

The irradiation processes were carried out using an illuminator Cole 41720-series of Palmer kipping a distance of 10 cm. between the surface of the irradiation source and the solution, the periods of irradiation time were varied at 25°C, under continuous agitation, with a maximum of the emission in UVA-sense 320–600 nm (3.3 mW/cm^2^, 45.575 Lux/seg) (the dose of the radiation was 4.5 J/cm^2^) measured with a Digital Radiometer UVX after 1 h of continuous illumination. For the determination of the photoinduced singlet oxygen generation and its quantum yield for the porphyrin **1**, 9,10-diphenylanthracene was used as a probe. This compound is an efficient scavenger of the ^1^O_2_ and its disappearance kinetics can be followed by UV-Vis spectrophotometry by its gradually decreasing absorbance at 374 nm [28–30]. The irradiation of **1** with visible light (> 420 nm) under oxygen was carried out in the presence of diphenylanthracene in chloroform. The irradiation at these wavelengths doesn’t affect the diphenylanthracene.

### Photosensitized oxidation of aromatic alcohols by compounds 1–4

A model reaction of photosensitized-oxidation was carried out for the oxidation of the α-naphthol to 1,4-naphthoquinone (equimolar 1.18 × 10^−4^ M). The porphyrin complexes **1–4** (5.0 × 10^−8^ M) were used as photogenerators of singlet oxygen on photosensibilization with UV-A.

First of all, the photostability of the substrate was inspected during 1 hour irradiation. A calibration curve was drawn. This emulates the reaction system with the descent of the sign of α-naphthol (λ_max_ = 295.74 ± 0.03 nm) as a function of the concentration of the singlet oxygen in the media. This calibration curve was traced using solutions at concentrations from 1.341 × 10^−4^ M (whose absorbance is 1 u.a. at λ_max_) to 9.44 × 10^−6^ M (whose absorbance is 0.1 u.a. at λ_max_). The calibration curve is traced by subtraction of the absorbances in λ_max_ between the reading of the more concentrated solution and the rest of them, generating in this way absorbance differences associated to differences in concentrations of α-naphthol, which can be related to instantaneous concentrations of singlet oxygen and of generated naphthoquinone.

### Energy Transfer

The studies on energy transfer and quenching of pyrenyl emission were carried out under argon in saturated dichloromethane solutions of compounds **1**–**4** using a spectrofluorophotometer LS-45 Perkin Elmer [21].

### Antibacterial photoactivity

The antibacterial assay was carried out using *Escherichia coli* (ATCC 8739) and their proliferation and viability were obtained by means of chemiluminescence using BacTiter-Glo Microbial Cell (Promega, USA). Compounds **1**–**4** were prepared in H_2_O/MDSO/Tw-80/NaCl (99.28:0.4:0.02:0.3), in a concentration range of 1.0 × 10^−4^ to 5.0 × 10^−6^ M. We took into account that different bacteria have different amounts of ATP per cell, and values reported for the ATP level in cells vary considerably. Factors that affect the ATP content of cells such as growth phase, culture medium, and presence of metabolic inhibitors, can influence the relationship between cell concentration and luminescence. The antibacterial photoactivity was carried out under irradiation with an illuminator LuzChem LZC 4V Photoreactor using 14 lamps with emission in UV-A (320–400 nm, 3.3 mW/cm^2^, 45.575 Lux/seg), keeping a distance of 10 cm between the lamp surface and the solution flask, varying the time periods of exposure at 37°C under continuous shaking.

The BacTiter-Glo Microbial Cell Viability Assay is a homogeneous method to determine the number of viable bacterial cells in culture based on quantification of ATP present. ATP is an indicator of metabolically active cells. The homogeneous assay procedure involves the addition of a single reagent (BacTiter-Glo® Reagent) directly on bacterial cells in LB Broth medium and followed by the measurement of luminescence. The luminescent signal is proportional to the amount of ATP present, which is directly proportional to the number of viable cells in culture.

The recorded luminescence signals (Luminescence (R.L.U: relative light units) represent the mean of three replicates for each measurement. The signal-to-noise ratio was calculated:

$$\text{S}:\text{N}=\frac{\text{mean}\;\text{of}\;\text{signal}-\text{mean}\;\text{of}\;\text{background}}{\text{standard}\;\text{deviation}\;\text{of}\;\text{background}}$$

A direct relationship (linear correlation) exists between luminescence measured with the BacTiter-Glo® Microbial Cell Viability Assay and the number of cells in culture over five orders of magnitude. Values represent the mean ± S.D. of 4 replicates for each cell number. The luminescent signal from 50 *Escherichia coli* cells is greater than three standard deviations above the background signal resulting from serum supplemented medium without cells. There is a linear relationship (r^2^ = 0.99) between the luminescent signal and the number of cells from 0 to 50,000 cells per well.

Factors that affect the ATP content of cells such as growth phase, medium, and presence of metabolic inhibitors, may affect the relationship between cell concentration and luminescence [31].

### Statistical treatment of results

At least three independent experiments were performed except where indicated otherwise. The quantification of results is expressed as a mean ± SD standard deviation (SD) is obtained from 3–4 observations. The level of significance accepted was *p* ≤ 0.05.

## Results

Yields of products and spectral data are summarized as follows:

### 5,10,15,20-Tetra(pyren-1-yl)porphyrin (TPyP, **1**)

C_84_H_46_N_4,_ MW: 1111.291 g/mol; 45.3% ± 0.2; MP: > 300°C. I.R. (KBr): 3448 (R-NH-R’), 1634 (C=C), 1099 (aromatics), 837, 797, 715 (aromatic C-H), 960 cm^−1^. MS (APCI): *m/z* 1110.37 (63%), 1111.36 (100%), 1112.35 (94%), 1113.40 (36%), 1114.38 (12%); (calcd. for {[M + H]+[M]}^+^: 1110,37(61%), 1111.38 (100%), 1112.38 (92%), 1113.39 (41%), 1114.39 (12%). Analysis, C_84_H_46_N_4_, calculated: %C 90.79, %H 4.17, %N 5.04; found: %C 90.82, %H 4.11, %N 5.00. ^1^H-NMR (300 MHz, CDCl_3_): δ = ppm; 9.40 (s, 2H, He), 8.70 (m, 4H, Ha), 8.50–8.10 (m, 24H, Hb), 7.63 (m, 8H, Hc), 7.50 and 6.80 (m, 8H, Hd).

### [5,10,15,20-Tetra(pyren-1-yl)porphyrinato(2-)-κ^4^N^21^,N^22^,N^23^,N^24^]nickel (NiTPyP, **2**)

C_84_H_44_N_4_Ni, MW: 1167.97 g/mol; 76.6% ± 0.3; I.R. (KBr): 2970, 1250, 1100, 1000, 800, 750 cm^−1^. MS (APCI): *m/z* 1168.71 (85%), 1167.68 (100%), 1111.35 (M^+^-Ni, 25%); (calcd. For [M+H]^+^) 1167.30 (100%), 1168.30 (90%). Analysis, C_84_H_44_N_4_Ni, calculated: %C 86.38, %H 3.79, %N 4.79; found: %C 86.42, %H 3.71, %N 5.00.

### [5,10,15,20-Tetra(pyren-1-yl)porphyrinato(2-)-κ^4^N^21^,N^22^,N^23^,N^24^]copper (CuTPyP, **3**)

C_84_H_44_N_4_Cu, MW: 1172.82 g/mol; 86.4% ± 0.3; I.R. (KBr): 3005, 1265, 1087, 1075, 795, 755 cm^−1^. MS (APCI): *m/z* 1177.77 (12%), 1176.77 ([M+H]^+^, 10%), 1175.09 (12%), 1174.10 (22%), 1173.47 (25%), 1172.48 (28%), 1171.49 (30%), 1147.39 (10%), 1111.50 (25%), 1006.31 (40%), 927.23 (100%); (calcd. For [M+H]^+^) 1176.00 (28%), 1175.20 (32%), 1173.20 (50%), 1172.00 (90%), 1171.29 (100%). Analysis, C_84_H_44_N_4_Cu, calculated: %C 86.02, %H 3.78, %N 4.77; found: %C 86.05, %H 3.69, %N 4.68.

### [5,10,15,20-Tetra(pyren-1-yl)porphyrinato(2-)-κ^4^N^21^,N^22^,N^23^,N^24^]zinc (ZnTPyP, **4**)

C_84_H_44_N_4_Zn, MW: 1174.67 g/mol; 87.4% ± 0,4; I.R. (KBr): 2987, 1280, 1099, 1012, 800, 748 cm^−1^. MS (APCI): *m/z* 1177 (22%), 1176.62 (50%), 1111.54 (M-Zn^+^, 100%); (calcd. 1176.79 (50%), 1111.38 (M-Zn)^+^, 100%). Analysis, C_84_H_44_N_4_Zn, calculated: %C 85.89, %H 3.76, %N 4.77; found: %C 85.80, %H 3.58, %N 4.60.

For the compounds **2** and **4** the ^1^H-NMR spectra were very similar. ^1^H-NMR (300 MHz, CDCl_3_): δ = ppm; 8.80 (m, 4H, Ha), 8.40–8.10 (m, 24H, Hb), 8.00 (m, 8H, Hc), 7.80 and 7.40 (m, 8H, Hd).

**Fig. 4.**
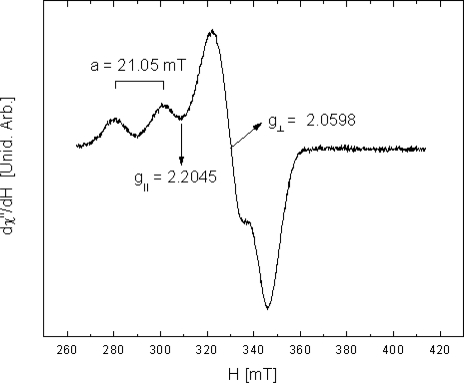
EPR TPyP Cu(II)spectra at 28°C in solid state.

In the specific case of the CuTPyP (**3**) a complete variation of the ^1^H-NMR spectrum in regard to the free base is evidenced. This is a consequence of the paramagnetic condition of the molecule due to the odd electron in the Cu(II) ion. It is well known that in the case of paramagnetic complexes the interactions of the generated magnetic field and the odd electron are considerably large and lead to chemical displacements much bigger that those associated to the ring current of the aromatic macrocycle. EPR spectra were registered by means of a Brucker EMX (Germany) at 28°C in solid state, EPR-TPyP Cu(II) (figure 4): 2.2045 〈*g*_‖_〉, 2.0598 〈*g*_⊥_〉, a=21.05 mT; and compared with some reported data [18]. These were compared as well with the tetraphenyl derivate TPPCu(II) commercially available from Sigma-Aldrich (St. Louis, MO, USA).

### Primary photophysical properties of compounds 1–4

The spectrophotometrical studies of compounds **1**–**4** were carried out using well-known concentrations in chloroform solutions. Since it is of vital importance, the fundamental step of any photochemical process is the absorption of a photon on behalf part of a photo-excitable molecule. In this way, the absorption spectra describe the regions of the UV-visible where these compounds absorb and hence are excited to higher energy states providing important data for the characterization of the synthesized photosensitizer.

The spectra show the characteristic maxima and the hypsochromic displacements. It is a prospective signal to characterize metallic complexes with metals of configuration d^6^–d^10^.

**Fig. 5.**
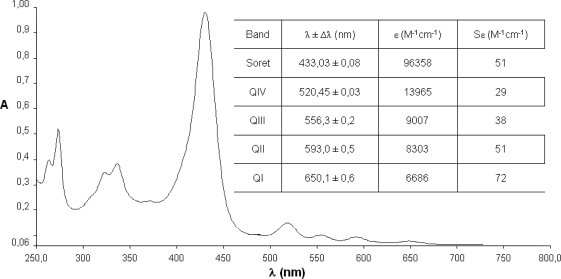
Absorption spectra of *meso*-tetra(pyren-1-yl)porphyrin (**1**).

**Fig. 6.**
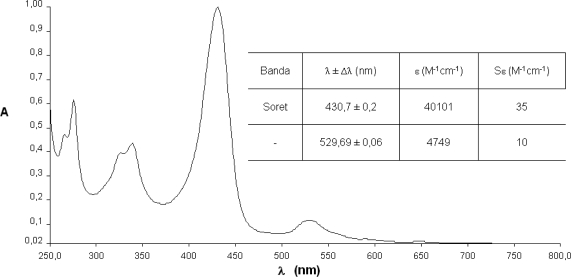
Absorption spectra of *meso*-tetra(pyren-1-yl)porphyrin-Ni(II) (**2**).

**Fig. 7.**
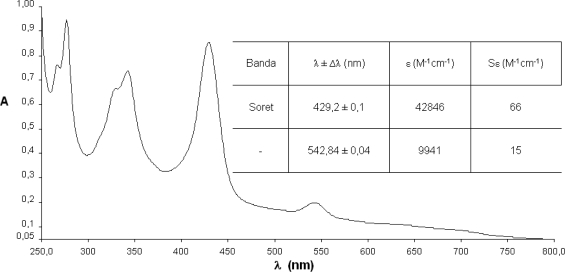
Absorption spectra of *meso*-tetra(pyren-1-yl)porphyrin-Cu(II) (**3**).

**Fig. 8.**
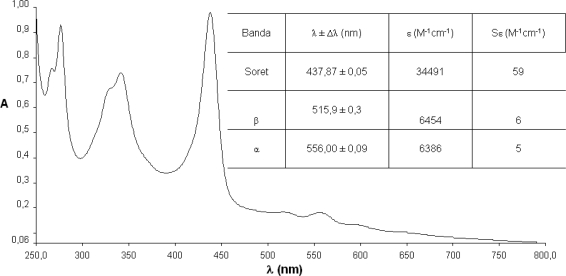
Absorption spectra of *meso*-tetra(pyren-1-yl)porphyrin-Zn (4).

In comparison with the free base (figure 5), the spectra of compounds **2**–**4**, lose the series of Q bands, which are substituted by one (NiTPyP – figure 6; CuTPyP – figure 7) or two maxima (ZnTPyP – figure 8). Additionally the molar absorptivities of the main bands are presented.

**Fig. 9.**
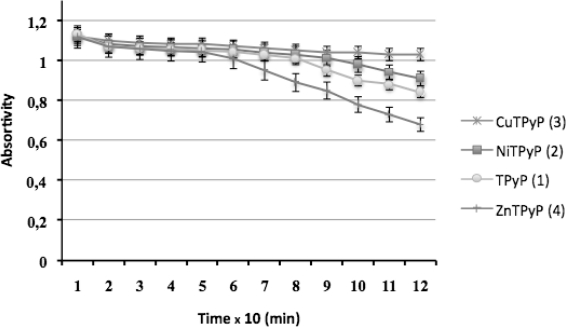
Photostability of compounds **1–4** under irradiation with UV-A light (absortivity of the soret band).

The study on the absorbance and fluorescence spectra of the synthesized compounds is important for a better understanding of their photochemical, photophysical characteristics and future photobiological studies. Thus, they are very useful for phototoxicity studies as well as their use as phototherapeutic agents or as photocatalysts for oxidation in organic reactions. These spectral data, for instance, are very important to select the appropriate wavelength for their detection and to compare their photobiological activity with other similar compounds. The spectrophotometrical studies carried out on photo-stability of compounds **1**–**4** under the experimental conditions of irradiation showed that they are photo-stable in all cases. The following by means of UV-vis spectrophotometry of long times of irradiation (up to 5 hours) showed no appreciable change in their spectra. Compounds **1**–**4** were appreciably photostable under UV-A (315–400 nm), but after 6 hours of irradiation a remarkable decrease of the intensity of the soret band was observed. The tendency to photostability under UV-A as follows: **3** > **2** > **1** > **4** (figure 9).

The compounds were very photostable under irradiation with visible light (400–700 nm) for more than 12 hours.

### Fluorescence quantum yields

The study of the phenomena of photophysical relaxation of an excited molecule (those that don’t imply changes in the molecular structure of a photosensitizer) are fundamental since at this stage are present: a) the different alternatives that the molecule can take to return to its ground state, b) which are the proposed routes for the return to take place and c) how we can manipulate the photochemical system to take advantage of these energetically favoured processes. It is possible to appreciate that the spectroscopic study of these emission processes allows inferring, qualitatively, the tendency to populate the triplet excited state T1, which is the first step to continue in the sequence of phosphorescent relaxation. It is possible in this way to generate a photochemical profile for each one of the metal complexes and from the free bases studied.

The solutions at well-known concentrations were saturated with argon to avoid the decline of the fluorescence signal as a product of the energy transfer for the generation of singlet oxygen. The following table shows the quantum yields of the synthesized compounds as well as the excitation and emission wavelength for each case.

Is it evident that the quantum yield of fluorescence from the Zinc derivative is larger than those derived from Nickel and Copper. Additionally, these three metallic complexes present a Φ_F_ considerably inferior than that of free base **1** (H_2_TPyP) = 0,226 ± 0,002 (λ_ex_= 422nm, λ_em_= 652nm) [28, 32, 33]. This is in agreement with that expected as a function from the spin-orbital coupling generated by the presence of the metallic ion in the structure of the photo-excitable molecule. This coupling can be described as the electromagnetic interaction between the spin magnetic moment of an electron in an orbital and a charged nucleus (+Z . e) as well as the coulombic field of this nucleus. This effect is proportional to the magnitude of *Z*, by means of which it is able to manipulate the system as to the inclusion of atoms of elevated atomic weight to favour a larger coupling.

The intrinsic effect on the system’s molecules is to facilitate the transitions between energy states of different multiplicity when minimizing the energy requirements that make these transitions processes quantically forbidden. The photosensitizers are a group of molecules which show an intrinsic effect of spin-orbital coupling in their molecular structure as consequence of a highly conjugated system, presence of heteroatoms of high atomic number, inclusion of transition metals, etc., which allow the intersystem crossing for the transition from an excited singlet state (generated by means of photo-excitation via sensitizer) to the lowest triplet excited state. The capacity to populate either states of different multiplicity and to facilitate the use of radiant energy as chemical energy is the reason that promotes the synthesis and characterization of new photosensitizers.

In the specific case of the metalloporphyrins, a central metal ion corresponds to the primordial factor of interference of the spin-orbital coupling as consequence of the multiple polarisable electron shells and a high nuclear charge density. It has been demonstrated that with the increase of Z value of the metal, the life time of the triplet state diminishes, Φ_F_ diminishes as well and the quantum yield of phosphorescence (Φ_P_) probability increases. This is in agreement with the drastic depression of Φ_F_ in the *meso*-tetra(pyren-1-yl)porphyrin (**1**) with the inclusion of a transition metal.

From the fluorescence quantum yields of compounds **2**–**4** we can infer that the effect of the spin-orbital coupling on the divalent ions of Ni and Cu is larger than that of the corresponding Zn (II) ion, although the fluorescence emission wavelength (λ_em_) for the complex ZnTPyP describes a bathochromic shift larger as a consequence of a decrease in the energy of the singlet state, product of the spin-orbital coupling, which favours the transition to the triplet state. This larger interference can be associated to the bond energy in Cu-N_4_(TPyP) as well as in Ni-N_4_(TPyP) which generates a stronger electronic interaction of both metals in the complexes, producing a major electronic stabilization and facilitating in this way the intersystem conversion S_1_ ∼> T_1._ In spite of this fact, the quantum yield of fluorescence doesn’t provide any good information regarding the phosphorescence or the energy transfer to the molecular oxygen to generate singlet oxygen. Therefore it is necessary to carry out specific assays to quantify the efficiency of each process. It is possible to state that for a Φ_F_ < 1 a molecular population exists in both excited states S_1_ and T_1_, and the decrease of the quantum yield of fluorescence in the case of the porphyrin derivates can be an indication of an increase in the efficiency of the singlet oxygen generation. However, as a consequence of the high spin-orbital coupling the possibility of favoring the non radiant intersystem crossing T_1_ to S_0_ exists inhibiting the energy transfer to oxygen, which implies the loss of energy of the excited state without the generation of singlet oxygen [34]. For this reason it is fundamental to determine the quantum yield of singlet oxygen production induced by the synthesized metalloporphyrins.

### Singlet oxygen generation

**Fig. 10.**
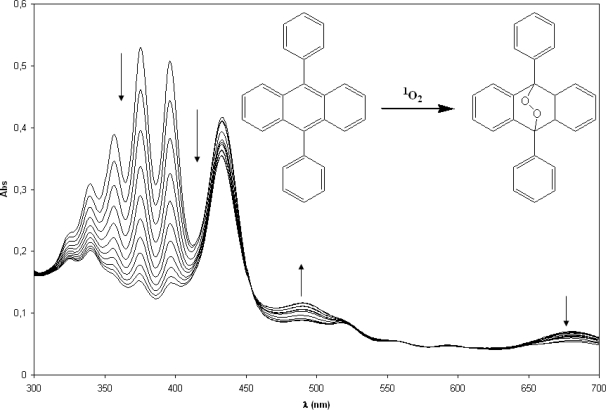
Disappearance of the 9,10-diphenylanthracene absorbance in presence of *meso*-tetra(pyren-1-yl)porphyrin (**1**) in CHCl_3_ / oxygenated media, irradiated with visible light. Registered spectra each 2 min.

Porphyrin **1** and its metallic complexes generate singlet oxygen efficiently when irradiated with visible light. The generation of singlet oxygen in the photolysis of the *meso*-tetra(pyren-1-yl)porphyrin (**1**) could be studied and quantified by means of the bleaching of the 9,10-diphenylanthracene (figure 10). The relative singlet oxygen quantum yield was 0.40 ± 0.02.

The methods to determine the absolute singlet oxygen quantum yield (Φ^1^O_2_) of compounds **1**–**4** imply the use of expensive equipments and specific detectors; for this reason in many cases indirect methods are used namely those in which the relative quantum yields are calculated in reference to a known pattern [35–37]. In the present investigation a method was applied based in the 1,4-cycloaddition reaction of ^1^O_2_ to diphenylanthracene (DPA).

The measurements were carried out after 30, 90 and 150 s. of UV-A irradiation, using the *meso*-tetraphenylporphyrin (H_2_TPP) as a control. The results for each one of the photosensitizers **1**–**4** are shown in the table 2.

### Study of the photoinduced oxidation of aromatic alcohols by compounds 1–4

**Fig. 11.**
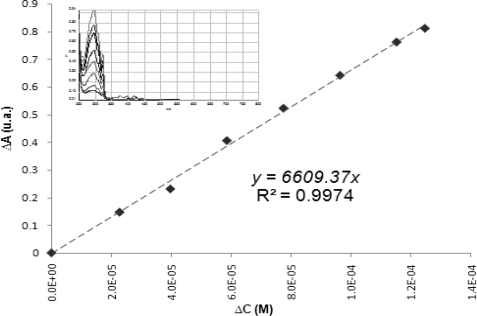
Calibration curve from quantification of naphthoquinone of the reaction between α-naphthol and ^1^O_2_, λ_max_= 295,7nm. Concentration solutions: from 1.341 × 10^−4^ to 9.44 × 10^−6^.

The first experiments revealed that all the compounds were photostable under UVA irradiation for one hour. Once inspected the photostability of the substrate, a calibration curve was drawn, which emulates the reaction system with the descent of the sign of α-naphthol (λ_max_ = 295.74 ± 0.03 nm) as a function of the concentration of the singlet oxygen in the media. The calibration curve is show in Figure 11.

The reaction yields after one hour of irradiation are shown in table 3. These data were determined as a function of the theoretical ideal yield, expecting a conversion of 100% of α-naphthol in the system.

The reaction yields are about 90% as evidenced for the system photosensitized by CuTPyP, which obviously represents a higher yield as compared to the other sensitizers. These values are in agreement with those expected as a function of the respective singlet oxygen quantum yields. These values were compared with the results published by Murtinho, et al.; for example, for 1,5-dihydroxynaphthalene reaction with ^1^O_2_ generated by *meso*-tetraphenylporphyrin (95%), *meso*-tetranaphthylporphyrin (84%) and *meso*-tetraphenanthrenylporphyrin (90%), considering that the time of irradiation in this system corresponded to two hours [38] while ours corresponds to one hours.

**Fig. 12.**
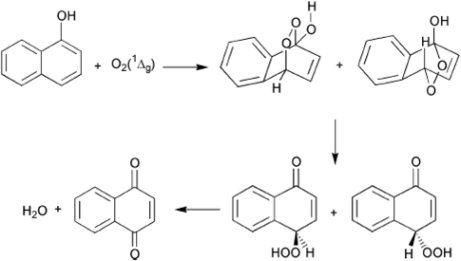
Mechanism of a type 1,4-photosensitized oxidation for the reaction of α-naphthol and ^1^O_2_ (generated by irradiation of compounds **1–4**) with the subsequent formation of naphthoquinone.

The mechanism proposed for this reaction postulates a 1,4-cycloaddition reaction with the subsequent formation of an endoperoxide. The electronic re-orientation and elimination of a water molecule takes place leading to the formation of the respective quinone (fig. 12).

### Energy transfer

In argon saturated dichloromethane solution compound **1** shows a significantly quenched pyrenyl emission (380, 397 nm, Φ = 0.17) and strong porphyrin fluorescence (655, 726 nm, Φ = 0.09) corresponding to approximately 76% of energy transfer from pyrene to porphyrin. In contrast, the porphyrin fluorescence of **2**, **3** and **4** are completely quenched as a consequence of the presence of a charge transfer state and only a weak pyrenyl luminescence is detected upon UV-excitation.

### Antibacterial photoactivity

**Fig. 13.**
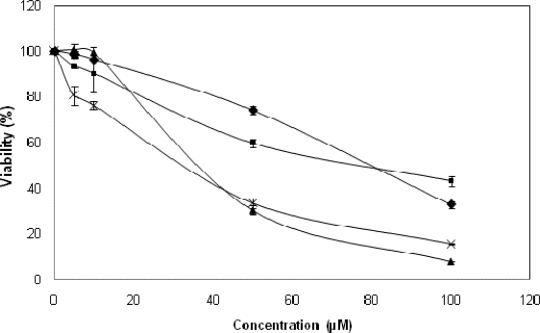
Cell viability assay (*E. coli*) in the presence of compounds **1** (•), **2** (×), **3** (▴), **4** (▪) at different concentrations. The result is based on quantification of the ATP present measuring by luminescence after 30 min irradiation each concentration.

Fig. 13 shows a comparative antibacterial action only photoinduced when compounds **1**–**4** were irradiated for 30 min in the presence of *E. coli* at different concentrations. It is clearly seen that all compounds exhibit a photoinduced dose-dependent photoinduced, where TPyPNi (**2**) presents the most pronounced effect. It should be noted that the results obtained in the absence of radiation showed no significant effects on the cell viability.

## Discussion

From the primary photophysical studies of compounds **1**–**4** it is important to denote that there exists a series of maxima between 250–350 nm which have been associated to electronic transitions product of the *meso*-substituted pyrenyl groups and that the B or Soret bands are preserved in the metalloporphyrins **2**–**4**, although there exists a small hypsochromic displacement in comparison with the free base.

The change in the symmetry of the molecule is evident when exchanging two protons in the macrocycle centre. These are displaced out of the plane by steric effects, by a metallic ion that occupies an equidistant position between the four pyrrolic nitrogens and forms interactions with all of them, generating in this way a considerably symmetrical molecule.

In the metalloporphyrins a degeneration of the ground and excited energy levels occurs, associated to the transitions of the pyrrolic nitrogens which are not present in the free bases, where two pairs of differentiated nitrogens exist. The transitions in the molecule as a consequence of the excitation of the nitrogenated groups observable in the range between 500 and 700 nm (the area of the Q bands in the case of the free bases, or of the bands α/β in the case of the metalloporphyrins), show a maximum of four possible electronic transitions for the free systems and only two possible transitions in the case of metallized porphyrinic systems (Figures 4–7) [30].

Singlet oxygen (^1^O_2_) is together with the hydroxyl radical (^·^OH) and the superoxide anion (^−^O_2_), one of the reactive species of oxygen photochemically generated. The peculiar singlet condition of its electronic configuration gives singlet oxygen a series of properties and chemical tendencies of reaction that take place with double bonds such as cicloadditions (conjugated or not) that in this way lead to the formation of cyclic peroxides of variable stability [2, 6, 39].

The present assay allows pointing out that the synthesized photosensitizers show a high photostability and are capable of generating singlet oxygen in high quantum yield, which allows the photooxydation of an aromatic alcohol with satisfactory results. This assay in homogeneous phase promises to encourage the investigation in new and better ways to improve the photochemical oxidation of organic substrates and to increase their number. In future investigations it will be possible to get better yields in heterogeneous systems varying the types and support methods.

The relevance of these oxidative agents is closely related to its ubiquitous presence both in biological systems as well as in synthetic chemistry. Furthermore, the easiness and effectiveness in its generation and the fact that its preparation does not involve an invasive activity in biological systems due to the short half life of singlet oxygen in PDT aqueous systems makes it a very acceptable oxidizing agent. On the other hand, from the industrial stand point it is preferred to other oxidizing agents because it is not offensive to the environment [3, 5, 6].

Due to the above mentioned advantages over other reagents it is important to carefully evaluate all the factors involved in the generation and efficiency of the synthesized porphyrinic photosensitizers. As it was mentioned before the ^1^O_2_ generation originates in an energy transfer from an excited triplet photosensitizer to the triplet ground state of oxygen in a triplet-triple annilation process. The latter is intimately associated to factors such as phosphorescence quantum Yield (Φ_P_), triplet state quantum yield (ΦT) and life time of the triplet state (τ_T_) which are affected by perturbations such as spin orbital coupling (SOC) and stereochemical effects due the transition metals coordinated to photo excitable organic moiety of the molecule.

The small decreasing tendency of the Φ^1^O_2_ values as a function of irradiation times in the four cases studied agrees with what is expected from the decrease in basal molecular oxygen concentration available as the reaction progresses, since the initial O_2_ saturation decreases in time. That is the case in spite of the fact that the process takes place in a closed system. In fact, that means that there is not a constant flow of O_2_ and the saturation of O_2_ in an organic solvent such as chloroform is considerably low (9.8 mM).

Due to all these factors it is easy to realize that only the singlet oxygen quantum yield (Φ^1^O_2_) at t= 30 s represents the efficiency for generating ^1^O_2_ in a system saturated of basal oxygen. This corresponds to a satisfactory approximation referred to the absolute Φ ^1^O_2_. Taking into account this approximation one can conclude that the quantum yield for generation of singlet oxygen is:

CuTPyP≫NiTPyP>TPyP>ZnTPyP

Finally, in order to determine the effect of the insertion of transition metals in the porphyrin nucleus on the quantum yield of singlet oxygen generated, it is important to compare Φ^1^O_2_ for the free base with that of the corresponding organometallic derivative. As a result for Φ^1^O_2_, τ= 30s gives the following new sequence:

$$\text{CuTPyP}\gg \text{NiTPyP}>{\text{H}}_{2}\text{TPyP}>\text{ZnTPyP}$$

From the new set of values it is evident that it is possible to infer that the process of energy transfer is by far more efficient for the chromophore CuTPyP tan that of its metallic analogs of Ni and Zn. The efficiency of the copper derivative is apparent since the generation of ^1^O_2_ in this case is faster and the consumption of basal oxygen takes place in a shorter time. In contrast, the consumption of basal oxygen in the presence of the other two metal chromophors (Ni and Zn) is slower, and allows for a competing deactivation process. In reference to the effect of the insertion of transition metals in the structure of the photosensitizer on the production of singlet oxygen as compared to that of the free base (table 2) one could infer that perturbations caused by insertion of Cu(II) and Ni(II) due to AEO (heavy atom effect) favor energy transfer to basal oxygen while inclusion of Zn increases radiationless transitions to the ground state S_0_. The possible reasons for this behavior are associated to stereochemical variations of the porphyrinic nucleus caused by volume departure from planarity due to the ionic radius of Zn which means deviation from planar symmetry and hence a decrease in energy transfer to ^3^O_2_ favoring the transition T_1_ to S_0_ (relaxation to ground state) [38].

Taking into account studies realized by other authors [40, 41] on the effect from the metals in phorphyrins on their electronic spectra, we can arrange that paramagnetic properties of copper (that increase population of the triplet state) seem to be a much plausible explanation of the difference in the singlet oxygen quantum yield generation between compound **3** and compounds **2** and **4**.

A comparative photoinduced antibacterial action was demonstrated in this work when compounds **1–4** were irradiated in the presence of *E. coli*. Initially in Figure 12, it is clear that porphyrin **2** has a more marked dose-dependent effect, because it shows that small changes in its concentration provide efficient inactivation. On the other hand, it is also clear that compounds **2** and **3** have an approximately 70% inactivation (reflected in a nearly 30% viability) at concentrations of 50 μM, while compounds **1** and **4**, have inactivation between 40 and 50% for the same concentration. The behavior observed in the photoinduced bacterial inactivation, is directly related to efficiency in the production of singlet oxygen (3> 2> 1> 4), thus confirming that photosensitizers capable of Type II reactions, have antibacterial characteristics.

## Conclusions

Inspection of the spectra may lead to reasonable conclusions. In the first place there exists an almost quantitative energy transfer between the excitation and emission maxima which implies that MTPyP are efficient photosensitizers when radiationless relaxation processes are favored. On the other hand, it may be noticed that when molecular oxygen is introduced in the system no fluorescence at all is detected in the three cases in point, which means that there exists a favorable situation for the formation of a triplet state sensitizer which in turn favors the formation of singlet oxygen.

Replacement of the phenyl rings with pyrene groups in the TPP has an appreciable effect on the absorption spectra which has an important incidence on photosensitization reaction, and hence on the generation of singlet oxygen by visible light making degradation of aromatic alcohols and bacterial inactivation more efficient.

## Authors’ Statement

### Competing Interests

The authors declare no conflict of interest.

## Figures and Tables

**Fig. 1.**
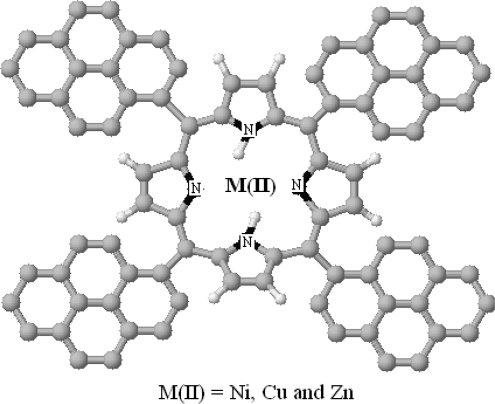
Structure of *meso*-tetra(pyren-1-yl)porphyrin (**1**). Nickel(II)-*meso*-tetra(pyren-1-yl)porphyrin (NiTPyP, **2**), Copper(II)-*meso*-tetra(pyren-1-yl)porphyrin (CuTPyP, **3**), Zinc(II)-*meso*-tetra(pyren-1-yl)porphyrin (ZnTPyP, **4**).

**Fig. 2.**
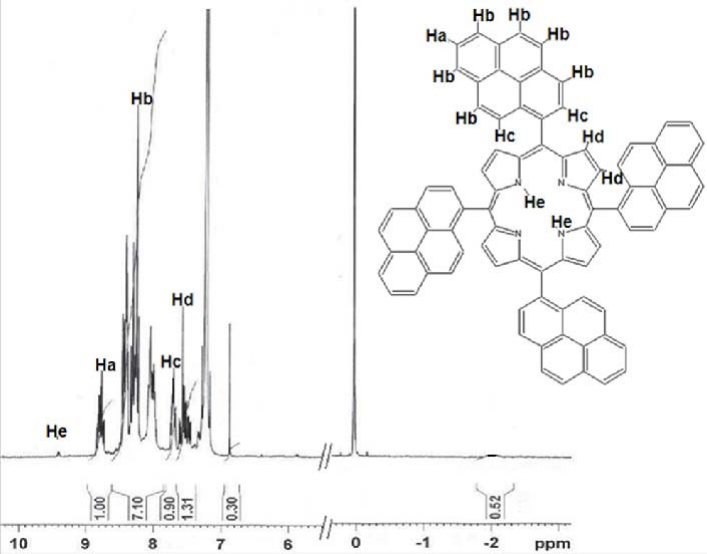
^1^H-NMR spectra of *meso*-tetrakis(1-pyrenyl)porphyrin (**1**).

**Fig. 3.**
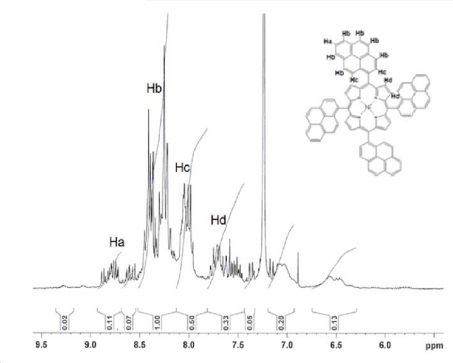
^1^H-NMR spectra of Ni- and Zn-*meso*-tetra(pyren-1-yl)porphyrin (**2**, **4**).

**Tab. 1.** Relative quantum yields (Φ_F_) of the synthesized compounds in chloroform and under argon.

**MTPyP**	**λ**_**ex**_ **(nm)**	**λ**_**em**_ **(nm)**	**Φ**_**F**_
**2** (NiTPyP)	348.92	656.12	0.132 ± 0.007
**3** (CuTPyP)	349.17	657.62	0.132 ± 0.007
**4** (ZnTPyP)	348.14	665.96	0.153 ± 0.008

**Tab. 2.** Singlet oxygen quantum yields (Φ^1^O_2_) for the complexes 1 to 4 (MTPyP: M = Ni, Cu, Zn) relative to the free base to (H_2_TPP) = 0.55 ± 0.01 [34] in CHCl_3_.

**Compound**	**t= 30s**	**t= 90s**	**t= 150s**
(**1**) H_2_TPyP	0.290 ± 0.02	0.280 ± 0.007	0.275 ± 0.005
(**2**) NiTPyP	0.320 ± 0.01	0.301 ± 0.006	0.297 ± 0.005
(**3**) CuTPyP	0.510 ± 0.01	0.490 ± 0.006	0.484 ± 0.005
(**4**) ZnTPyP	0.203 ± 0.008	0.194 ± 0,005	0.188 ± 0.004

**Tab. 3.** Yields of the phosensitized oxidation of α-naphthol with ^1^O_2_, after one hour of UVA irradiation.

**Compound**	**Yield (%)**
H_2_TPyP	79.0 ± 0.3
**(2)** NiTPyP	85.0 ± 0.3
**(3)** CuTPyP	92.0 ± 0.3
**(4)** ZnTPyP	80.1 ± 0.2
